# Updated dosimetric comparison of HDR interventional radiotherapy including ^60^Co sources versus VMAT and electron beam therapy for NMSC

**DOI:** 10.1007/s00411-026-01258-z

**Published:** 2026-10-09

**Authors:** Sidra Sajjad, Muhammad Shahban, Enrico Rosa, Bruno Fionda, Humera Mehmood, M. Basim Kakakhel, Luca Tagliaferri, Marco De Spirito, Elisa Placidi

**Affiliations:** 1 Pakistan Institute of Engineering and Applied Sciences (PIEAS), Islamabad, Pakistan; 2 Nuclear Medicine, Oncology and Radiotherapy Institute (NORI), Islamabad, Pakistan; 3 Fondazione Policlinico Universitario Agostino Gemelli, IRCCS, Rome, Italy; 4 Università Cattolica del Sacro Cuore, Rome, Italy

**Keywords:** non-melanoma skin cancer, high-dose-rate interventional radiotherapy, brachytherapy, cobalt-60, volumetric modulated arc therapy, electron beam therapy

## Abstract

Non-melanoma skin cancers (NMSCs), primarily basal cell carcinoma and squamous cell carcinoma, are the most common malignancies worldwide and frequently arise on the scalp, where complex curvature and anatomical constraints make treatment planning challenging. While surgery remains the standard approach, radiotherapy, particularly high-dose-rate interventional radiotherapy (HDR-IRT, modern brachytherapy), has become an important alternative for patients who are not ideal surgical candidates. Previous dosimetric analyses have demonstrated that ^192^I HDR-IRT provides superior target coverage and dose conformity compared with external beam techniques such as volumetric modulated arc therapy (VMAT) and electron beam therapy (EBT). However, the reproducibility of these findings across centres and radioactive sources, including ^60^Co, has not been systematically validated. This study aimed to independently validate prior dosimetric results by independently replanning the same cohort of 25 patients with scalp NMSC using ^60^Co HDR-IRT. The objective was to assess whether the dosimetric advantages of HDR-IRT remain consistent when different operators, institutions, and isotopes are employed, and to compare ^60^Co HDR-IRT with VMAT and EBT under identical clinical conditions. A retrospective dosimetric comparison was conducted using the original CT datasets from 25 patients previously analyzed with ^192^Ir HDR-IRT. At an independent institution, new plans were generated for ^60^Co HDR-IRT, VMAT, and EBT using the same contours, applicator geometry, prescription doses, and V_95%_ constraints. HDR-IRT plans were constructed in Oncentra Brachy v4.6 based on TG-43 formalism; VMAT and EBT plans were generated in Eclipse TPS v16.1 using 6 MV photons and 6 MeV electrons, respectively. Dosimetric parameters (V_90%_, V_95%_, V_100%_, D_90%_, D_mean_, D_max_) and brain D2cc values were extracted from DVHs. Statistical comparisons were performed with the Mann–Whitney U test (*p* < 0.05). All techniques provided comparable V_90%_ (> 99%) and V_95%_ values, ensuring consistent baseline coverage. However, HDR-IRT using Co-60 demonstrated significantly higher V_100%_, D_90%_, and D_mean_ values than VMAT and EBT across all prescription groups (*p* < 0.05). This reflects the proximity of the source to the target and the steep dose gradient inherent to brachytherapy. VMAT produced the most homogeneous dose distributions but at the expense of slightly reduced high-dose coverage. EBT performed adequately for small, flat lesions but showed pronounced limitations in large or curved anatomies, with increased variability and occasional hotspots in the brain. Brain D_2cc_ doses remained comparable across modalities (*p* > 0.05), confirming adequate protection of critical structures. This external validation confirms that HDR-IRT retains its dosimetric superiority over VMAT and EBT even when replanned by different operators, at a different institution, and using an alternative isotope (^60^Co instead of ^192^Ir). The findings demonstrate that HDR-IRT is a robust and reproduciblemodality for scalp NMSC, capable of achieving excellent target coverage and rapid dose fall-off without compromising organ-at-risk sparing. These results strengthen the evidence supporting HDR-IRT as a reliable option for treating superficial scalp cancers across diverse clinical settings.

## Introduction

Non-melanoma skin cancers (NMSCs) are the most prevalent malignant tumours diagnosed globally, affecting for example 2 to 3 million people across the United States annually (Skowronek 2015). According to the International Agency for Research on Cancer (IARC), nearly 20 million new cases of cancer were reported worldwide in 2022, with approximately 0.18 million in Pakistan. There were 9.7 million cancer-related deaths (0.7 million with NMSC), including 0.11 million in Pakistan (Bray et al. 2024). The incidence of NMSC correlates directly with age, fair skin, and latitude. The risk of skin cancer among the white population increases nearly twofold for every 10 degrees of latitude closer to the equator (Zablow et al. 1992). Basal cell carcinoma (BCC) and squamous cell carcinoma (SCC) together account for 99% of NMSCs, with SCC making up 25% and BCC 75%. Their incidence rates continue to rise annually but have a low mortality rate. Although BCC and SCC, also known as keratinocyte carcinomas (KCs), have low metastatic potential, they significantly impact the quality of life (Safigholi et al. 2015).

Tumours frequently develop on the scalp. Anatomically, the scalp begins from the occipital region and spreads to the forehead, reaching the eyebrows. It extends from the temples to the cheekbones on the sides (Emmanuel J. K. Adu 2013). Most scalp tumours are benign, while 20% are malignant, with SCC and BCC being the most prevalent. Scalp lesions often go undetected in the early stages because they can be concealed by hair. Patients usually seek medical attention only when the lesions become painful, ulcerated, bleed, release an offensive or purulent fluid, or interfere with their ability to perform daily tasks (Prodinger et al. 2018).

Scalp lesions can be treated using several treatment methods, including surgery, radiotherapy and systemic therapy (Lomas et al. 2012; Rogers and Coldiron 2013; Zaorsky et al. 2017). Surgical excision and Mohs micrographic surgery (MMS) are the most used treatments for skin cancer due to their minimal local recurrence rates (< 5%). However, these methods have limitations such as their invasive nature and high annual costs (Delishaj et al. 2015). Surgical procedures in sensitive areas may result in nerve damage, movement-limiting scarring, and poor cosmetic outcomes, with delayed or non-healing surgical wounds occuring more likely in elderly than in young patients with various medical comorbidities (Delishaj et al. 2016; Garcovich et al. 2017). Due to advancements in external beam radiotherapy (EBRT) and interventional radiotherapy (modern brachytherapy), these modalities have been adopted as an alternative treatment option for patients with high surgical risks (Taylor et al. 2021).

Iridium-based HDR-IRT is effective for treating skin tumours (Fionda et al. 2023, 2024), using surface mold HDR-IRT. This technique uses molds made from flexible materials, such as silicone or polymethyl methacrylate, that are designed to fit the surface of the cancer. Radioactive sources are subsequently inserted into the mold, ensuring an even distribution of radioactive dose across the entire tumour volume. This method is typically administered at a high dose rate (HDR) of less than 12 Gy/h. It has advantages over traditional radiotherapy techniques, including lower recurrence rates, effective local tumour control, better cosmetic outcomes, and a shorter treatment duration for NMSCs (Alam et al. 2011). Various studies indicate that HDR-IRT has gained prominence in the last decade for treating NMSCs, achieving local tumour control rates of 90% to 98% and resulting in favourable cosmetic outcomes in 98% of treated patients. HDR-IRT is characterized by a minimally invasive approach and improved tolerability for elderly patients, who may struggle with lengthy surgical procedures or may have comorbidities that negatively impact wound healing and cosmetic results (Taylor et al. 2021).

Volumetric modulated arc therapy (VMAT) is another advanced technique used for treating NMSCs. VMAT offers advanced beam modulation and more conformal dose distribution than conventional three-dimensional conformal radiotherapy (3D-CRT), delivering a more homogeneous dose to target while reducing irradiation of surrounding normal tissues.

This minimizes dose to the surrounding OARs and prevents hot and cold spots, reducing the risk of scarring and cancer recurrence (Spelman et al. 2022). VMAT necessitates planning and robust quality assurance measures to ensure the best possible results.

EBT is also a viable option for treating superficial tumours. This modality provides a unique dose distribution profile that is advantageous for lesions requiring limited penetration depth. EBT is well-suited for flat, small, and medium-sized tumours. However, its effectiveness decreases on large, complex, curvy, or rounded surfaces. Notably, a decrease in plan quality was found to depend more on target curvature than on tumour size (Buzurovic et al. 2021).

In clinical practice, the selection among HDR-IRT, VMAT, and EBT depends strongly on lesion characteristics. HDR-IRT is particularly suitable for superficial tumours with irregular or highly curved surfaces, especially when located near critical structures such as eyes and head and neck region where rapid dose fall-off is desirable (Rosa et al. 2026). Electron beam therapy is generally appropriate for small, flat lesions with limited thickness, where uniform superficial dose can be achieved. Finally, VMAT may be considered for larger or more complex volumes requiring homogeneous dose distribution, but may involve greater low-dose exposure to surrounding tissues.

In a previous study (Placidi et al. 2024) a retrospective dosimetric comparison between ^192^Ir HDR-IRT, VMAT, and EBT was performed in 25 patients with scalp NMSC.

This study demonstrated that HDR-IRT achieved superior target coverage and dose conformity while minimizing deep-tissue exposure compared to external beam techniques. The present work represents an update and independent validation of that study. Specifically, it was conducted independently by another institution, using the same CT datasets, but replacing the ^192^Ir source with Cobalt-60 ^60^Co) for HDR-IRT planning.

Cobalt-60 has a longer physical half-life (5.27 years) than ^192^Ir (74 days), which makes it particularly advantageous in centres with limited resources. However, its clinical and dosimetric equivalence in surface HDR-IRT for NMSC has not been thoroughly evaluated.

Consequently, the aim of the present study was to validate the reproducibility of the previously reported dosimetric findings by performing an independent reanalysis of the same patient cohort, using HDR-IRT plans generated with an alternative radioactive source and by a different institution.

## Materials and methods

A retrospective analysis was conducted at the Nuclear Medicine, Oncology, and Radiotherapy Institute (NORI), Pakistan, using anonymized CT scans from 25 patients with scalp NMSC included in the original publication. All patients had been initially treated with ^192^Ir HDR interstitial brachytherapy (IRT) using a Freiburg applicator. For the present update, ^60^Co HDR-IRT plans were generated using the same applicator geometry and clinical parameters. Each plan was reconstructed and optimized in Oncentra Brachy v4.6 (Elekta) following the TG-43 formalism (Nath et al. 1995).

The same imaging datasets were imported into Varian Eclipse TPS v16.1 to create VMAT and EBT plans, replicating the previous workflow. VMAT plans used 6 MV photons, while EBT plans employed 6 MeV electron beams with customized applicators and shielding blocks. All plans were optimized to achieve V_95%_ coverage between 95% and 100%.

For each technique, dose–volume histogram (DVH)-derived dosimetric parameters were extracted, including V_90%_, V_95%_, and V_100%_ (percentage of the target volume receiving at least 90%, 95%, and 100% of the prescribed dose, respectively), D_90%_ (dose delivered to 90% of the target volume), D_mean_ (mean dose), D_max_ (maximum dose), and brain D2cc (minimum dose received by the most irradiated 2 cm³ of the brain). Statistical comparisons were performed using the Mann–Whitney U test, with statistical significance set at *p* < 0.05.

Tumour volume ranged from 1 cm^3^ to 142.2 cm^3^, with an average clinical target volume (CTV) of 24.6 cm^3^. On average, ten plastic catheters were used per patient (range: 4–27). Three dose regimens were prescribed: Group A (patients 1–11) received 54 Gy in 18 fractions, Group B (patients 12–16) received 50 Gy in 20 fractions, and Group C (patients 17–25) received 40 Gy in 8 fractions.

**Fig. 1:**
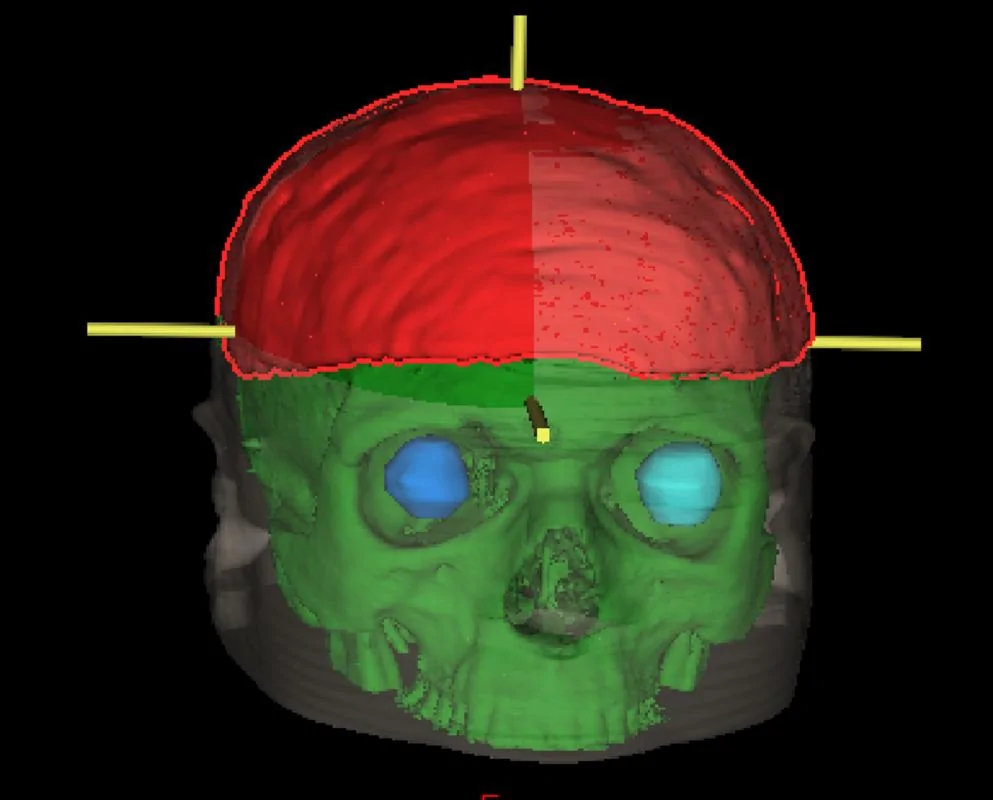
Extensive scalp lesion precluding the feasibility of an electron beam therapy (EBT) plan (clinical target volume (CTV) = 142.2 cm^3^)

Four patients—#5 (77.84 cm^3^), #6 (42.39 cm^3^), and #9 (81.6 cm^3^) from Group A, and #17 (142.2 cm^3^) from Group C—were not suitable for EBT due to large and curved CTVs. In particular, patient #17 (Fig. 1) showed significant tumor height variation and complex scalp curvature, making it difficult to achieve homogeneous coverage with standard 6 MeV electrons without exceeding acceptable brain dose constraints. In such geometries, dose uniformity becomes highly sensitive to surface irregularities and setup conditions, thereby limiting the feasibility of electron planning.

For ^60^Co HDR brachytherapy, the CTV and brain were contoured by a radiation oncologist. Catheters were manually reconstructed slice by slice, and dwell positions were activated along each catheter covering the CTV with a 3 mm margin. Initial dwell times were normalized to achieve adequate V_95%_ coverage, followed by manual graphical optimization aimed at maximizing V_100%_ and D_90%_ while limiting high-dose hotspots (D_max_) and minimizing dose to adjacent organs at risk. No inverse planning algorithm was used. Optimization was performed by experienced radiation oncologists and medical physicists according to institutional clinical practice.

Dose calculations followed TG-43 (Nath et al. 1995), and manual optimization minimized doses to organs at risk (OARs).

VMAT plans were generated with 1–2 full arcs and 1–3 partial arcs, a 0.5 cm bolus, and appropriate collimator angles. Optimization applied ICRU Report 50 target limits and QUANTEC-based OAR constraints (Jones 1994; Bisello et al. 2022). Brain dose limits were set to 60 Gy (2 Gy/fraction), 54.5 Gy (2.5 Gy/fraction), 50 Gy (3 Gy/fraction), and 37.5 Gy (5 Gy/fraction); eye limits were 45 Gy, 40.9 Gy, 37.5 Gy, and 28.1 Gy, respectively.

EBT plans used 6 MeV beams, a 0.5 cm bolus, varied couch/gantry angles, and applicators (A6, A10, A15) with shielding blocks. Dose calculations employed the AAA algorithm (Ulmer et al. 2005). Both VMAT and EBT plans maintained V_95%_ coverage between 95% and 100%, ensuring OAR doses remained within acceptable limits.

All plans were generated by medical physicists with more than five years of experience in skin brachytherapy and EBT planning. Planning procedures followed institutional clinical protocols.

Finally, target parameters (V_90%_, V_100%_, D_90%_, D_mean_, D_max_) and OAR metrics (D_2cc_ for brain and eyes) were compared to assess coverage and sparing. Statistical analysis used the Mann–Whitney U test (*p* < 0.05).

## Results

### Target coverage

The target coverage data are presented in Table 1. V_95%_ was kept constant to ensure consistency, and V_90%_ also remained the same (> 99%). However, V_100%_ and D_90%_ exhibited noticeable differences, with HDR-IRT showing higher values due to its close source placement. EBT plans showed intermediate values as electrons deposit most of their energy near the surface. Overall, brachytherapy offered superior target coverage.

**Table 1:** Comparative dosimetric parameters of target coverage across three different modalities and groups

	^60^Co IRT	VMAT	EBT
***Group A***			
**V**_**90%**_ **(%)**	99.5 ± 0.5	99.9 ± 0.1	99 ± 1.2
**V**_**95%**_ **(%)**	98.2 ± 0.9	98.7 ± 1.3	98.1 ± 1.5
**V**_**100%**_ **(%)**	96.7 ± 2.8	50.8 ± 3.1	87.7 ± 12.3
**D**_**90%**_ **(%)**	102 ± 2.4	97.7 ± 0.6	101.2 ± 2.8
***Group B***			
**V**_**90%**_ **(%)**	99.8 ± 0.1	99.9 ± 0.1	99.6 ± 0.9
**V**_**95%**_ **(%)**	99.0 ± 0.6	99 ± 0.6	99.6 ± 0.5
**V**_**100%**_ **(%)**	92.9 ± 3.1	52.5 ± 1.5	76 ± 11.8
**D**_**90%**_ **(%)**	101.8 ± 2.6	97.9 ± 0.2	99 ± 1
***Group C***			
**V**_**90%**_ **(%)**	99.6 ± 0.5	99.8 ± 0.4	99.3 ± 1
**V**_**95%**_ **(%)**	98.6 ± 1.4	98.3 ± 2.6	98.3 ± 1.7
**V**_**100%**_ **(%)**	94.3 ± 4.7	52 ± 2.4	85.8 ± 11.4
**D**_**90%**_ **(%)**	104.1 ± 4.4	97.5 ± 0.8	100.9 ± 2.9

All dose parameters are expressed as percentage of the prescribed dose. V_90%_, V_95%_, and V_100%_ represent the percentage of clinical target volume (CTV) receiving at least 90%, 95%, and 100% of the prescribed dose, respectively. D_90%_ represents the minimum dose received by 90% of the CTV. Values are reported as mean ± standard deviation. IRT – interventional radiotherpay ; VMAT – volumetric modulated arc therapy; EBT – electron beam therapy

### CTV dose distribution

**Fig. 2:**
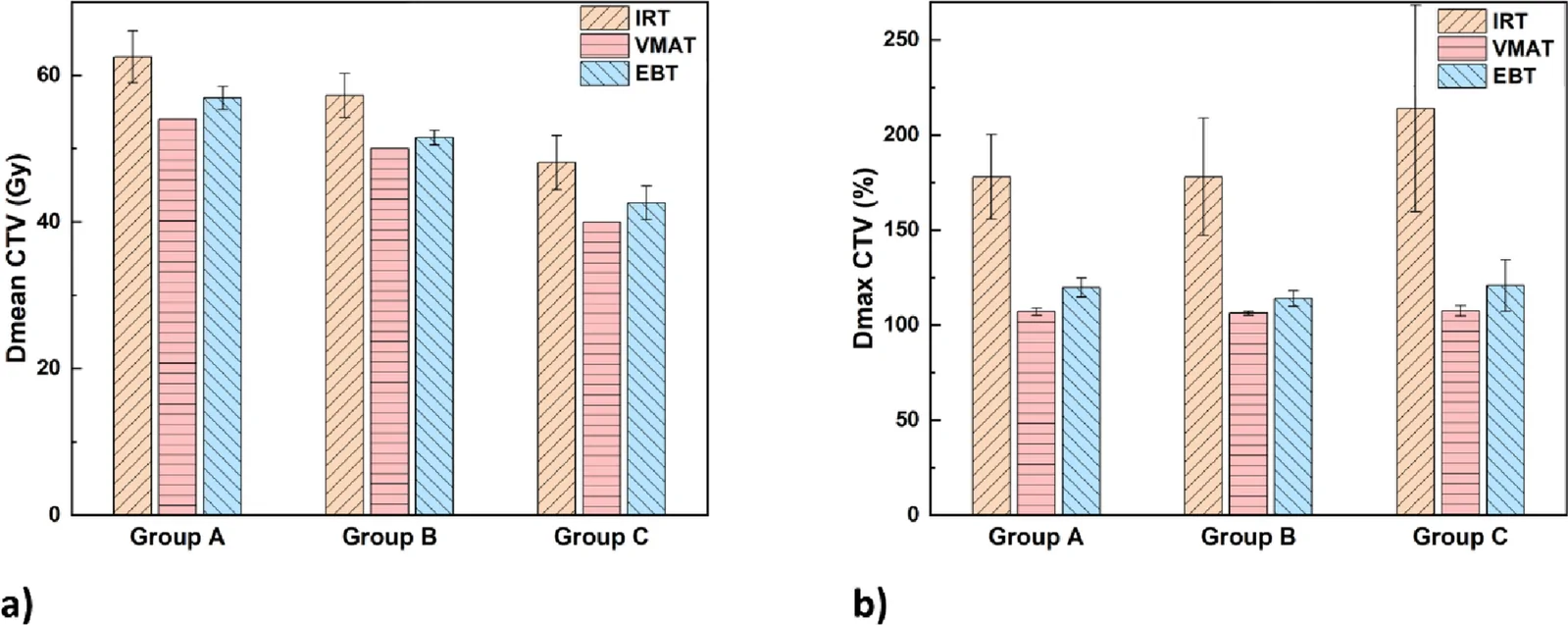
Dosimetric comparison of clinical target volume (CTV) across three treatment modalities (^60^Co interventional radiotherapy (IRT), volumetric modulated arc therapy (VMAT) and electron beam therapy (EBT) and prescription groups (A, B, and C): (a) Mean dose and (b) Maximum dose; error bars represent standard deviations

Figure 2 depicts CTV dose distributions in terms of mean and maximum doses across three treatment methods. IRT delivered the highest D_mean_ and D_max_ values, attributed to its steep dose gradient and proximity to the radioactive source. EBT showed moderate dose values due to its buildup and limited penetration, while VMAT had the lowest values, reflecting its uniform dose delivery. A statistically significant difference (*p* < 0.05) in mean and maximum doses was observed amongst the three modalities. For instance, IRT D_mean_ values exceeded by 7–9 Gy for VMAT and 5–6 Gy for electrons. Similarly, IRT exhibited the highest D_max_, exceeding VMAT by 71–107% and electrons by 58–94% across various groups. These findings highlight IRT’s capability to deliver a more concentrated and localized dose to the CTV than the other modalities, due to the proximity of the radioactive source to the target, an advantage for treating superficial tumors.

### OAR dose analysis

Radiation exposure to critical organs, such as the brain, was assessed across all modalities where dose constraints are essential to minimize toxicity.

**Fig. 3:**
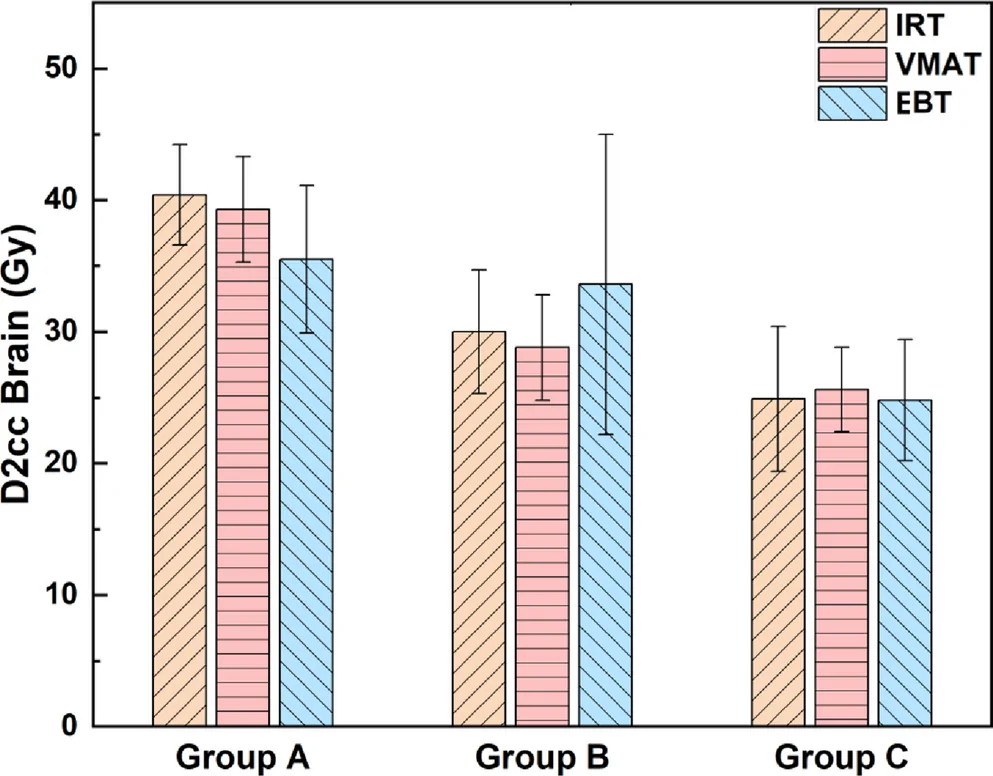
Assessment of D_2cc_ brain across three prescription groups (A–C) and techniques (^*60*^*Co* interventional radiotherapy (IRT), volumetric modulated arc therapy (VMAT) and electron beam therapy (EBT). D_₂cc_ represents the minimum dose received by the most irradiated 2 cm³ of the brain. Error bars represent standard deviations

D_2cc_ analysis of the brain, as illustrated in Fig. 3, revealed that all treatment techniques provided comparable brain dose, with no statistically significant differences (*p* > 0.05). However, EBT showed greater variability, as five patients’ plans produced hotspots in the brain while attempting to achieve a V_95%_ between 95% and 100%. In a lesion with complex topology, achieving such a high level of target coverage often leads to increased doses to the brain. Therefore, a trade-off between adequate CTV coverage and OAR protection is sometimes necessary to stay within clinical dose limits.

## Discussion

The main objective of this work was to validate and extend the dosimetric findings previously reported, in which ^192^Ir HDR-IRT was compared with VMAT and EBT for the treatment of scalp non-melanoma skin cancer.

By reanalyzing the same cohort of 25 patients at an independent institution using a different radioactive source (^60^Co), this study aimed to test the reproducibility and robustness of HDR-IRT dosimetry across centres and planning conditions.

It should be noted that formal inter-operator variability analysis was not performed; therefore, these findings should be interpreted as evidence of institutional reproducibility rather than strict operator independence.

With regard to isotope selection, it is important to clarify that this study did not aim to establish full physical equivalence between ^192^Ir and ^60^Co sources. Rather, the objective was to assess whether the dosimetric trends previously observed with ^192^Ir could be reproduced when the same applicator geometry and TG-43-based calculation formalism were applied using ^60^Co. Under these standardized planning conditions, similar coverage patterns were observed. Therefore, the present findings suggest reproducibility of dosimetric behaviour across these two isotopes within the constraints of the adopted calculation model.

Overall, the present analysis confirmed the findings of the original study: HDR-IRT consistently achieved superior target coverage and dose conformity compared with VMAT and EBT.

As shown in Table 1, ^60^Co -based HDR-IRT achieved the highest V_100%_ and D_90%_ values, while maintaining comparable V_95%_ and V_90%_ across all techniques. These results were statistically significant (*p* < 0.05) and fully aligned with the previous ^192^Ir-based results, demonstrating that the dosimetric advantage of HDR-IRT is not dependent on the specific radioactive isotope or on operator experience.

The superior performance of HDR-IRT can be attributed to the proximity of the radiation source to the target volume, producing a steep dose gradient and allowing for optimal surface coverage with rapid dose fall-off in deeper tissues. As demonstrated in a recent study (Placidi et al. 2023), HDR-IRT could be further optimized and refined through personalized approaches, especially for the treatment of small lesions.

From a radio-physical perspective, the dosimetric differences observed in this study can be explained by the intrinsic characteristics of each modality. In HDR-IRT, the radioactive source is positioned in close proximity to the target, resulting in a steep dose gradient governed by the inverse square law. This allows high surface dose deposition with rapid fall-off in deeper tissues, which is particularly advantageous for superficial scalp lesions. In contrast, VMAT relies on external photon beams that exhibit a build-up region and greater sensitivity to bolus positioning and setup uncertainties, potentially affecting surface dose accuracy. Electron beam therapy, while characterized by finite penetration depth, is highly sensitive to surface curvature and tissue inhomogeneity, which may compromise dose homogeneity in complex scalp geometries.

VMAT achieved the most homogeneous dose distribution but required larger low-dose volumes to achieve acceptable coverage, particularly in irregular scalp geometries.

EBT, while effective for small and flat lesions, showed limited conformity and increased variability for large or curved targets, confirming the challenges of electron planning in complex scalp topologies.

OAR analysis revealed that the brain D_2cc_ doses were comparable across all techniques (*p* > 0.05), confirming that HDR-IRT provides excellent target coverage without compromising normal tissue sparing. This observation, which mirrors the results from the ^192^Ir study, underscores the inherent dosimetric balance achieved by HDR-IRT; highly localized dose escalation to the tumour with efficient protection of critical structures. Importantly, this validation was performed independently, with different planners and software environments, yet yielded consistent outcomes. Such reproducibility emphasizes the robustness of HDR-IRT as a treatment modality and supports its reliability for clinical implementation across institutions.

Adequate target coverage parameters such as V_100%_ and D_90%_ have been associated with improved local control in skin brachytherapy. Conversely, excessive high-dose regions (D_max_) at the skin level may correlate with increased risk of late toxicity, including ulceration or sub-optimal cosmetic outcomes. Therefore, the balance between coverage and avoidance of excessive hotspots is clinically relevant in NMSC management. Although this study was limited to dosimetric evaluation, the observed differences should be interpreted in light of these established clinical correlations.

Within the limitations of TG-43-based planning and identical applicator geometry, the dosimetric advantage appeared reproducible across ^192^Ir and ^60^Co sources.

Although the study was based on dosimetric simulations rather than clinical outcomes, the close agreement with prior data provides strong support for HDR-IRT’s role in the treatment of superficial scalp lesions.

This study has limitations. First, it represents a retrospective dosimetric comparison without direct clinical outcome data. Second, dose calculations were performed using the TG-43 formalism, which does not account for tissue heterogeneities or applicator attenuation. Third, formal inter-operator variability analysis was not performed; therefore, reproducibility across planning teams should be interpreted within the context of independent institutional replanning rather than strict operator independence. Prospective studies including clinical endpoints are warranted to confirm whether the observed dosimetric advantages translate into improved patient outcomes.

## Conclusions

This external validation study confirmed the reproducibility and robustness of high-dose-rate interventional radiotherapy for the treatment of scalp non-melanoma skin cancer.

When the same patient datasets were independently replanned at another institution using a different radioactive source (^60^Co), HDR-IRT outperformed VMAT and EBT in target coverage, conformity, and dose fall-off.

These findings demonstrate that the dosimetric characteristics of HDR-IRT are consistent and reproducible when independently replanned at a different institution by a separate planning team.

The results strengthen the evidence that HDR-IRT represents a reliable, source-independent, and reproducible technique across institutions and planning teams, capable of achieving precise dose delivery and excellent target coverage while maintaining protection of surrounding normal tissues.

In summary, this study validated the robustness of HDR-IRT as a treatment modality for NMSC and confirms that its dosimetric superiority over external beam techniques remains stable across institutions, planners, and radioactive sources.

## Data Availability

The data presented in this study are available from the corresponding author upon reasonable request.
